# Targeting dePARylation for cancer therapy

**DOI:** 10.1186/s13578-020-0375-y

**Published:** 2020-01-29

**Authors:** Muzaffer Ahmad Kassab, Lily L. Yu, Xiaochun Yu

**Affiliations:** 1 Department of Cancer Genetics & Epigenetics, Beckman Research Institute, City of Hope, Duarte, CA 91010 USA; 2 Westridge School, 324 Madeline Dr., Pasadena, CA 91105 USA

**Keywords:** PARG, ADP-ribosylation, dePARylation, DNA damage response, Cancer therapy

## Abstract

Poly(ADP-ribosyl)ation (PARylation) mediated by poly ADP-ribose polymerases (PARPs) plays a key role in DNA damage repair. Suppression of PARylation by PARP inhibitors impairs DNA damage repair and induces apoptosis of tumor cells with repair defects. Thus, PARP inhibitors have been approved by the US FDA for various types of cancer treatment. However, recent studies suggest that dePARylation also plays a key role in DNA damage repair. Instead of antagonizing PARylation, dePARylation acts as a downstream step of PARylation in DNA damage repair. Moreover, several types of dePARylation inhibitors have been developed and examined in the preclinical studies for cancer treatment. In this review, we will discuss the recent progress on the role of dePARylation in DNA damage repair and cancer suppression. We expect that targeting dePARylation could be a promising approach for cancer chemotherapy in the future.

## Overview

DePARylation is the process that removes ADP-ribose (ADPR) signals from various proteins during cellular stresses conditions such as DNA damage response (DDR) [1]. During DDR, ADPR moieties are attached to the substrate proteins by various poly(ADP-ribose) polymerases (PARPs) with PARP1 and PARP2 catalyzing the predominant function [2–4]. The ADP-ribosylation can just be a single ADP-ribose (mono-ADPR/MAR/MARylation) or a long chain of repetitive ADPR units (poly-ADPR/PAR/PARylation) [5, 6]. The PARylation signals anchor additional proteins containing PAR-binding motifs (PBMs) to the sites of damaged DNA. Thus ADP-ribosylation functions as an important post-translational modification trafficking proteins to the site of damaged DNA for DNA repair thereby helping in maintaining genomic stability [1, 3, 7, 8].

DNA damage activates PARP1/2 that in turn generates covalently attached MAR/PAR chains onto themselves (auto-PARylation) and other acceptor proteins (trans-PARylation) utilizing NAD^+^ as an ADP-ribose donor and generating nicotinamide as a byproduct. PARylation modulates the function and structure of the modified proteins. The modified proteins, in turn, recruit additional proteins involved in DDR to the damaged loci [2, 9]. PARylation is a reversible modification, and consequently, this modification is terminated and cellular homeostasis is attained. The removal of PAR chains is mainly attained due to the hydrolysis of these polymers by poly(ADP-ribose) glycohydrolase (PARG) [10, 11]. However, PARG cannot remove the terminal ADP-ribose and thus the complete removal of the PARylation signals requires additional enzymes [12]. The additional hydrolases include TARG1 terminal ADP-ribose protein glycohydrolase (TARG1), macrodomain containing proteins MacroD1/D2 and recently discovered ADP-ribose-acceptor hydrolases ARH1/3 [1, 13–15].

Therapeutic perturbation of the PARylation/dePARylation processes has successfully demonstrated the selective killing of cancerous cells. Most notably, PARP1/PARP2 inhibitors (PARPi) are actively used in the clinical treatments of familial breast and ovarian cancers with partial DDR defects [16, 17]. PARPi suppresses PARP1/PARP2 function, which in turn prevents an optimal DDR [18–20] thereby inducing cell death. However, unfortunately, like other chemo-drugs, cancers resistance to PARPi has emerged [21–23]. Recent countermeasures to overcome this resistance have focused on the development of inhibitors against dePARylation proteins and more specifically against PARG. Since PARG is responsible for reversing the majority of PARylation, anti-PARG inhibitors (PARGi) have demonstrated the promising potential for killing cancerous cells at an efficacy equitant to PARPi [24, 25]. PARGi like PARPi has shown synthetic lethal phenotype in cells deficient in DDR proteins. Besides, PARG being a monogenic protein unlike the redundant PARP enzyme family, a higher degree of specificity could be achieved with PARGi [26]. Here, we review our current understanding of the dePARylation proteins and focus on the recent advancement of exploiting dePARylation proteins in anti-tumor therapies.

## PARylation in DNA damage repair

PARylation is a transient and reversible protein post-translational modification that modulates the structural and functional properties of the acceptor proteins during a wide variety of biological processes including DDR, cell stress, transcription, immune response, aging and cell death [3, 4, 27]. However, the well-characterized function of PARylation is its role in the regulation of DNA repair signaling. PARylation is catalyzed by a large family of proteins (17 members in total, from PARP1–PARP4, PARP5a–PARP5b and PARP6–PARP16) known as poly(ADP-ribose) polymerases (PARPs). All PARPs share a huge degree of homology with the founding PARP family member PARP1. PARP1 and PARP2 are dominant PARP family enzymes in the cells and act as the primary sensors of DNA damage [2, 3]. PARP1 is the most abundant PARP protein in a cell (1–2 million molecules/cell) accounting for 90% of cellular PARylation, while PARP2 accounts for the remaining 10% [5, 28, 29]. Apart from PARP1 and PARP2, additional PARP proteins contribute a minor fraction of PARylation or MARylation, PARP9 and PARP13 lack enzymatic activity [30]. Upon DNA damage, PARP1 physically attaches to the damaged DNA through its N-terminal zinc-finger domains and the interaction activates the C-terminal catalytic domain [31, 32]. The activated PARP1 then hydrolyzes NAD^+^, resulting in the polymerization of ADPR units on PARP1 itself as well as a huge number of proteins involved in DDR [33]. PARP1 itself is heavily autoPARylated during PARP1 activation [34, 35]. During polymerization, NAD^+^ is hydrolyzed into ADPR and nicotinamide is generated as a side product. The first ADPR is covalently attached to the acceptor proteins usually through an ester linkage [36]. PARylation could involve either the attachment of a single or multiple ADPR moieties. Repeated units of ADPR are polymerized into long PAR chains (*O*-glycosidic bonds) which could attain linear and/or branched conformation. A single PAR chain can polymerize up to 200 residues in each polymer and the branches are incorporated after every 20 to 50 residues [37]. The vast majority of ADPR attachment primarily involves glutamate, aspartate, serine [38], arginine and lysine [39] residues in acceptor proteins. The attachment thus involves an *O*-glycosidic bond for glutamic acid, aspartic acid and serine, while an *N*-glycosidic bond is formed on arginine and lysine [40, 41].

PAR chains due to the negatively charged phosphates of ADPR bring a lot of anionic charges to the damaged chromatin and the negative charges alter the chemical and biological properties of the acceptor proteins. The acceptor protein of PARylation includes histones (H1, H2A, and H2B) [41, 42], DNA protein kinases [43, 44], p53 [45], Ku complex [3, 46], DNA glycosylase 8-oxoguanine glycosylase 1 (OGG1) [47], PCNA [48], RUNX [49], etc. Since DNA is negatively charged; charge repulsion between PAR and DNA modulates the chromatin structure at the damaged loci. Besides, proteins containing PAR binding motifs/domains are recruited to the DNA damage site by the PAR signal itself. These downstream proteins include XRCC1 [50], DNA ligase III [51], CHFR [52] and once recruited, these proteins promote protective DNA damage repair. Thus DNA-damage associated PAR signals act as a docking signal and a scaffold on which a huge number of DDR proteins are assembled which favors efficient and optimal DDR [3, 7].

PARP1 enzymatic activities are required for all forms of DNA damage including mismatch repair, base excision repair, SSB repair and DSB repair [28]. PARP2 enzymatic activities are however limited to BER and restarting blocked replication forks [53]. However, our recent studies indicate that PARP2 plays an important role in branched PAR chain synthesis. The frequency of branching was decreased by more than half in PARP2 knockout mice which was rescued by wild type PARP2. We observed that PARP2 mediated branching was initiated by the PAR chain interaction with N-terminus of PARP2 and the branching PAR was important for the recruitment of histone removal proteins (e.g. APLF) during DNA damage repair [5].

Although PARP1 and PARP2 are crucially required for maintaining genomic stability, mice lacking either protein are viable, although these mice are hypertensive to DNA damaging agents [3, 54]. This discrepancy is attributed to the high redundancy of these two PARP proteins during embryonic development. Thus, the lack of one PARP protein can be complemented by the other. However, synthetic lethality is achieved when both PARP enzymes (e.g. PARP1 an PARP2 [55]) and proteins involved in DDR are inhibited [56]. This property is clinically exploited and used by anti-PARylation inhibitors in cancer therapy and will be discussed in detail later in this review.

## The role of dePARylation in DNA damage repair

**Fig. 1:**
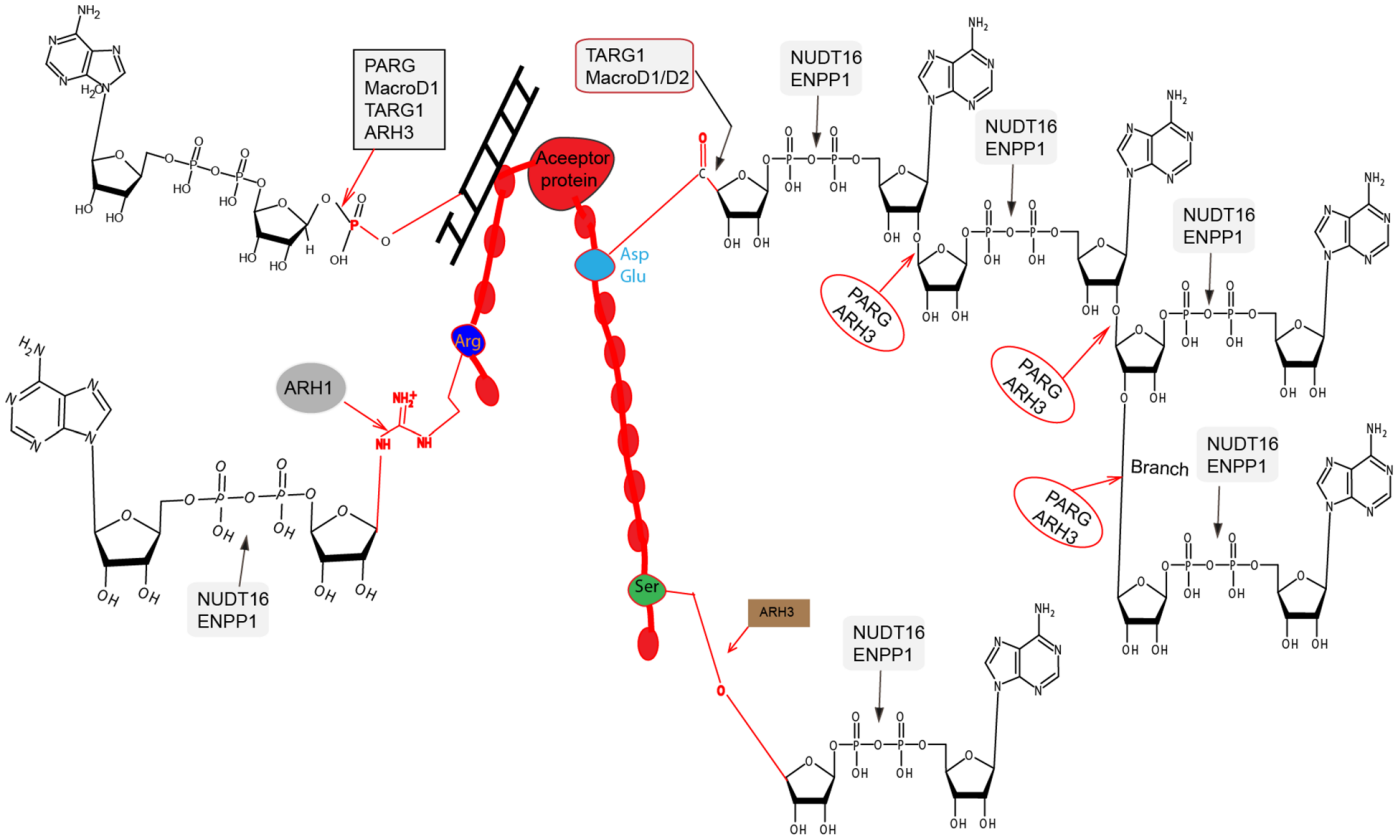
Schematic diagram showing the proteins involved in dePARylation. The acceptor protein is shown as beads on a string. The dominant amino acids involved in PARylation (i.e. aspartic acid and glutamic acid are shown light blue, arginine is shown in dark blue, serine is shown in light green). A dsDNA helix is and MAR moiety attached to it is shown in black. The bonds hydrolyzed by different dePARylation proteins are shown. A linear and branched PAR chain is shown attached to aspartic acid and glutamic acid

PARylation and MARylation, like other biological modifications, are precisely regulated. The de-polymerization terminates the ADP-ribosylation-associated signaling cascade and steady-state is achieved. PARylation recruits DDR proteins near the damaged loci and dePARylation facilitates their deposition onto the damaged site. Failure to remove the PAR signal will result in the trapping of DDR proteins at the vicinity of the damaged DNA and causing cells hypersensitive to DNA damage [24, 57]. The rapid hydrolysis of PAR happens almost immediately after PAR synthesis is achieved. These hydrolases include PARG, TARG1, MacroD1, MacroD2, ARH1 and ARH3 (Fig. 1). These proteins contain a highly conserved macro domain fold hydrolyzes the glycosidic bond among ADPR units or between ADPR and protein residues [13–15]. PARG is the dominant enzyme involved in PAR chain removal and this activity is only weakly observed in TARG1 and ARH3. TARG1, ARH1, ARH3, MacroD1 and MacroD2 are involved in the removal of proximal ADPR/MAR. Recent studies have implicated two other pyrophosphatases Nudix Hydrolase 16 (NUDT16) and Ectophosphodiesterase/nucleotide phosphohydrolase (ENPP) that may digest the phosphor-diester bond in ADPR during PAR metabolism.

### PARG

The majority (~ 90%) of the cellular PAR chains are digested by the catabolic enzyme PARG [58, 59]. Alternative splicing of a single PARG gene product generates five isoforms that have variable size, cellular distribution and activity [60, 61]. PARG is recruited to the PAR locations by PCNA which is facilitated by acetylation of lysine (K409) on PARG [27, 62]. PARG acts as both endo and exo-glycohydrolase [63], and is well suited to hydrolyze the *O*-glycosidic bonds between the ADPR units of PAR; however, as mentioned earlier, the terminal ADPR is linked to an acceptor protein via an ester bond and consequently resists PARG mediated hydrolysis [12, 64]. Nevertheless, PARG is critically important for cellular function and loss of PARG causes embryonic lethality in mice [65]. Thus, unlike PARP1/PARP2 deletion which can be tolerated by cells to a certain extent, PARG-associated function is required for cell viability. Our recent studies confirmed the role of PARG in DNA damage repair. We observed that shRNA mediated knockdown of PARG was associated with defective DNA single-stranded and double-stranded break repair by trapping DDR factors on PAR chains at the damaged loci [24]. Thus, precisely regulated PARP1 and PARG activities promote optimal response to cellular stress conditions.

### TARG1

TARG1 is an 18 7kd Macro domain-containing protein that predominantly hydrolyzes mono-ADPR from aspartate and glutamate. TARG1 is primarily recruited to a damaged site by PAR signals wherein it cleaves the last ADPR moiety from the side chains of aspartate and glutamate residue following PARG-mediated dePARylation [66, 67]. Thus, one major function of TARG1 is to complete the PAR removal process once PARG direst the rest of PAR chains. In addition, TARG1 has weak PAR removal function probably due to the removal of the whole PAR chain directly from aspartate and glutamate [66].

### ARH1 and ARH3

ARH family enzymes resemble dinitrogenase reductase-activating Glycohydrolase (DraG) family enzymes that mediate nitrogen fixation in bacteria [68]. ARH1-3 proteins are identical in size (39 kDa) and share similar primary sequences. ARH1 and ARH3 hydrolases primarily act on MAR moieties on the acceptor proteins. ARH1 is mainly involved in the hydrolysis of the *N*-glycosidic bond formed between arginine and ADPR [69]. ARH3 has the strongest hydrolytic activity within the ARH family, which like PARG can hydrolyze PAR chains of the acceptor proteins. However, unlike PARG, which lacks terminal hydrolase activity, ARH3 can remove MAR moieties as well. Recent studies have implicated ARH3 in the hydrolysis of the glycosidic bond between serine and ADPR [70]. Serine-ADP-ribosylation is emerging as an abundant form of protein ADP-ribosylation on DNA damage response proteins primarily catalyzed by PARP1 and PARP2 [71, 72]. ARH2 binds to ADPR but has no reported activity on either MARylated or PARylated proteins. Thus ARH1 and ARH3 proteins, along with TARG1 are required for a complete reversal of PARylation post-PARG mediated digestion.

### MacroD1 and MacroD2

These proteins share similar Macro domain fold (also found in PARG and TARG1) probably emerging from gene duplication during evolution. MacroD1 and MacroD2 like TARG1 are required to remove the proximal ADPR from the Asp and Glu residues of the acceptor proteins. They can also function as dominant MAR hydrolases in the cells [13, 15]. Recent studies suggest that MacroD1 is also involved in the deMARylation of dsDNA [73].

### NUDT16 and ENPP

NUDT16 is a member of Nudix superfamily hydrolases found across all living organisms encompassing archaea, bacteria and eukaryotes. These family enzymes are primarily involved in the digestion of pyrophosphate containing substrates like dNTP, nucleoside di- and triphosphates, etc. Interestingly, both PAR chains and MAR can act as a substrate for NUDT16 [74]. However, due to the phosphatase nature of NUDT16 catalysis, ribose-5′-phosphate is retained on the acceptor proteins and phosphoribosyl-AMP is released. Thus complete reversal of PARylation/MARylation cannot be achieved by NUDT16 and the proteins required for removing the NUDT16 signature sequence are not known [9, 75]. ENPP is a recently characterized PAR/MAR phosphodiesterase and like NUDT16 catalysis, ENPP mediated catalysis is characterized by retention of ribose-5′-phosphate on acceptor proteins at the PAR/MAR attachment site [76].

## PARP inhibitors in cancer treatment

PARylation is responsible for both initial sensing of DNA damage and the recruitment of DNA damage response proteins to the damaged site. Consequently, disruption of this crucial cellular signal is associated with the accumulation of DNA lesions, which causes cell death. However, as discussed earlier, despite its critical role in maintaining genomic integrity, PARP1/PARP2 knockout does not induce lethality. This is attributed to the redundant function of PARP proteins as well as multiple DNA damage repair pathways. However, cells deficient in the alternative DNA repair pathways/proteins undergo apoptosis upon PARP1/PARP2 inhibition. This feature is known as synthetic lethality, a phenomenon in which cells with one defect i.e. either a mutation in DDR or PARP inhibition can survive, but a combination of the two together causes cell death [77–79].

PARPi mediated synthetic lethality has been exploited in clinical cancer treatment. PARPi including olaparib, rucaparib, niraparib, talazoparib act by both inhibiting PAR formation as well as by blocking the PARP1 release from the damaged DNA. These inhibitors are PARP1/2 competitive inhibitors and compete with the cellular NAD^+^ for binding to PARP1 [80]. Moreover, the inhibitors trap PARP1/2 on the sites of damaged DNA forming PARP-DNA complexes and the stalled replication forks cause cell death [57]. Olaparib was the first PARPi to receive FDA approval for the treatment of advanced ovarian cancer with BRCA mutations in 2014. Over the past few years, olaparib has been extended and FDA approved in treatments of other cancers including triple-negative breast cancer and pancreatic cancer with BRCA mutations.

PARP inhibitors have developed into promising and potent therapeutic strategies against a wide variety of cancers. However, unfortunately, like with other cellular therapies, PARPi resistance has emerged in the clinic [21, 81]. Apart from increased drug efflux, these resistance mechanisms involve reverent mutations in BRCA1/2 genes. These reverse mutations produce normal protein and the cells switch to normal HR in presence of PARPi. Besides, PARP1 protein itself may be lost from the cells under prolonged PARPi treatment. Moreover, cells may partially restore PARylation by inactivating PAR digesting enzyme PARG [82]. An additional mechanism of resistance involves the inactivation of critical DDR proteins like 53BP1, SLFN11 [83], REV7, EZH2, BRD7 [84], EMI1 [85], etc. Recent studies have indicated that PARPi ovarian cancer cells can attain resistance to PARPi due to enhanced microhomology-mediated end joining attributed to increased expression of ALDH1A1 [86] and due to decreased m^6^A levels on FZD10 mRNA which in turn activates Wnt signaling pathway [87].

## Developing PARG inhibitors

DePARylation is equally important for proper cell function as PARylation. Complete coordination of the two processes is essential for proper DNA damage response. We and others have shown that dePARylation is not merely an antagonistic process of PARylation in the context of DNA damage repair. Instead, dePARylation is an immediately downstream step of PARylation. The function of PARylation is to mediate the recruitment of DDR factors to the proximity of DNA lesions, whereas dePARylation releases these DDR factors from PAR chains, so that these factors can be loaded at exact DNA lesions for repair. Suppression of dePARylation traps DDR factors onto the PAR chains, thus impairs SSB and DSB repair [24]. Moreover, therapeutic inhibition of PARP enzymes is compounded due to the presence of multiple PARP isoforms. Since, mice lacking PARP1 or PARP2 are viable, double knockouts of PARP1 and PARP2 leads to the death of the mice [55]. This phenotype of the double knockout mice suggests that the two proteins play a redundant role and in the absence of one PARP (e.g. PARP1) the function can be compensated by other PARP protein (e.g. PARP2). Accordingly, during therapeutic inhibition of one PARP enzyme, uninhibited enzymes may compensate for the lost PARP protein.

**Fig. 2:**
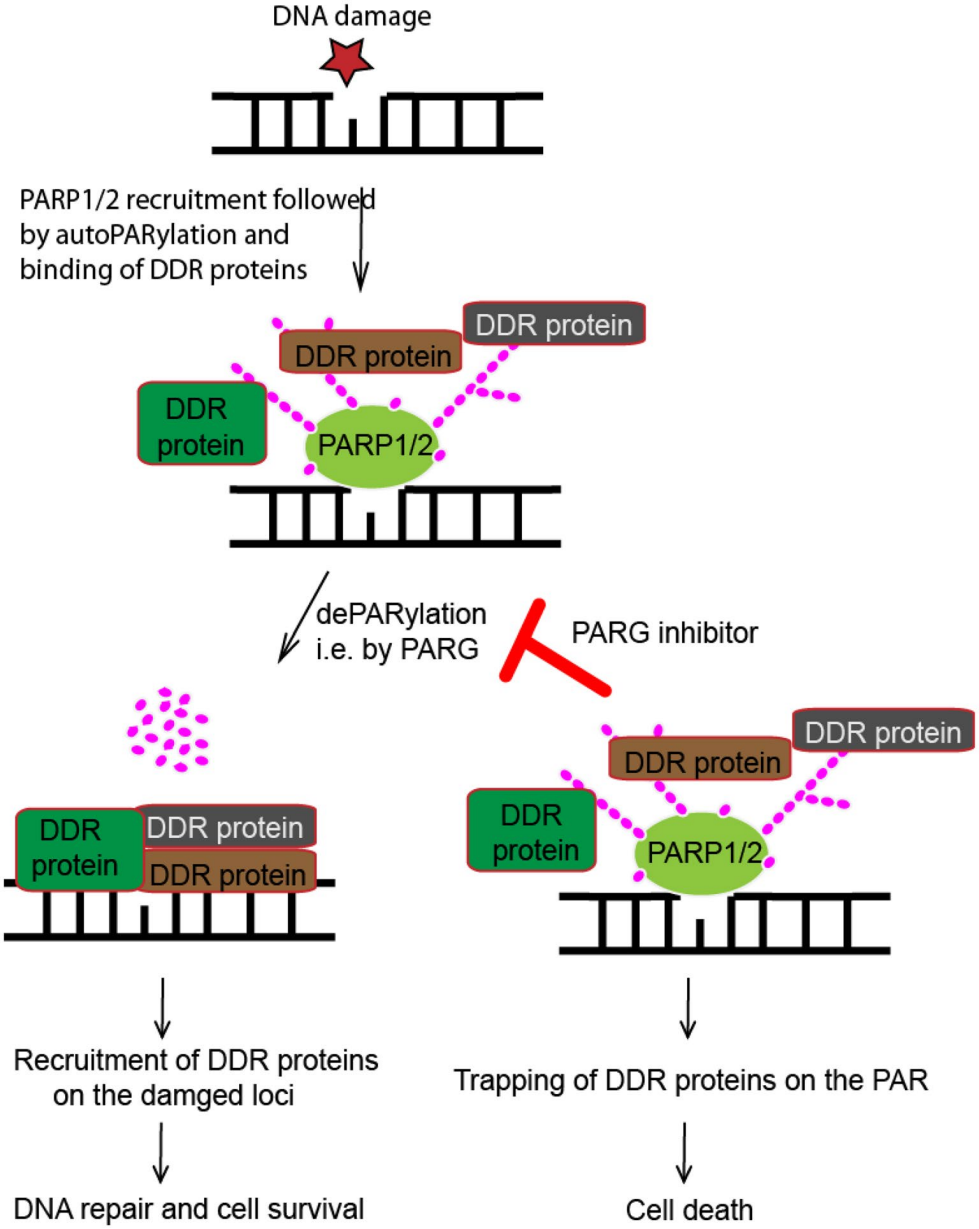
Schematic diagram showing the effect of dePARylation inhibitors on DDR. A damaged (star) DNA is shown on the top. PARP proteins (PARP1/2) are recruited to the site where they undergo PARylation and MARylation. The PAR/MAR moieties recruit DDR proteins to the vicinity of the damaged site. DePARylation (i.e. by PARG) digests the PAR chains, relives the PARP proteins and loads the DDR on the damaged site. DePARylation inhibitors trap the DDR on the PAR chains thereby preventing DDR and leading to cell death

Targeting of dePARylation may circumvent some of the problems associated with PARPi resistance. PARG unlike multiple PARP proteins is monogenic and does not share its dePARylation function. Consequently, higher potency and specificity could be achieved with PARG inhibition [1, 3] (Table 1 and Fig. 2). In addition, PARPi resistance involves loss of PARP1 itself from the resistant cells. However, a similar PARG loss is unlikely due to the cell lethal phenotype of the PARG deficient cells.

**Table 1:** PARG inhibitors and their drawbacks

Inhibitor	Activity	Limitations and IC_50_	Cancer type/model	References
Intercalating molecules, e.g. proflavine, ethidium bromide, ethacridine	Bind PAR and resist PARG mediated hydrolysis	Not effective in-vitro and cell impermeable, IC_50_ > 7 μM	Ex-vivo	[11, 88]
GPI16552 and GPI 18214	Same as above	Not effective in-vitro, IC_50_ > 1.7 μM	Colon tissue mice	[11, 89]
Tannins e.g. Nobotanin K	ADPr analogs i.e. bind PAR and resist PARG	Low cell permeability, IC_50_ > 0.3 μM	Cell line	[11, 90]
Salicylanilides	Bind PARG and inhibit dePARylation	Not effective in-vivo and non-specifically (inhibit PARP1), IC_50_ > 12 μM	Cell line	[92]
RBPIs	Block PARG mediated PAR hydrolysis	Low specificity and less potency, IC_50_ > 2.9 μM	Cell line	[93]
PDD00017273	Replication fork stalling and low DNA double stranded break repair	Low metabolic activity, IC_50_ > 25 nM,	Cell line	[25, 96]
COH34	Binds PARG catalytic site and traps DDR proteins	IC_50_ = 0.37	Mice	[24]

The first generations of PARG inhibitors were DNA intercalating polyaromatic molecules such as proflavine, ethidium bromide and ethacridine. These inhibitors directly bind the PAR chain thereby blocking PARG mediated hydrolysis of the polymer [88]. The potency of intercalators was enhanced with the introduction of two more PARG inhibitors GPI 16,552 and 18,214 and the inhibitors were presented as potent PARG inhibitors against ant-inflammatory protection [89]. The next class of PARG inhibitors was tannins (e.g. Nobotanin K) [90] and ADP-ribose analogs (e.g. Adenosine diphosphate (hydroxymethyl)pyrrolidinediol (ADP-HPD) [91]. These inhibitors were effective in-vitro but lacked cell permeability which would prevent their use on cells.

The first cell-permeable PARGi were identified via a high throughput screening known as salicylanilides which were originally used in fungicide treatments. These inhibitors had an added advantage of inhibiting both PARG as well as PARP1 [92]. Yet another class of synthetic inhibitors based on Rhodamine was developed known as rhodanine-based PARG inhibitors (RBPIs). These inhibitors (e.g. RBPI-1) were specific to PARG with no effect on ARH3 or PARP1 [93]. The inhibitors were more potent than salicylanilides exhibiting high specificity and cell permeability [92]. However, therapeutic testing of these inhibitors was associated with low specificity, less potency and thus with low druglikeness [25].

## Application of PARG inhibitors in cancer treatment

As mentioned earlier, PARG inhibition can circumvent the majority of problems associated with PARP1 inhibition. Additionally, recent studies have indicated that increased PARG expression is associated with higher incidences of breast cancers as well as cellular transformation and invasion in vivo [94]. Moreover, PARG suppression has been implicated to prevent lung cancer in PARG^+/−^ mice treated with benzo(a)pyrene by stabilizing the expression of Wnt ligand [95]. These observations make PARG a perfect target for cancer chemotherapy. To address the problems associated with previously known PARG inhibitors, D.I. James and colleagues performed high-throughput screening and developed a cell-permeable PARG inhibitor PDD00017273. PDD00017273 caused dose-dependent inhibition of PARG and significant PARG inhibition could be achieved at low concentrations (0.3 μM). When breast cancer MCF7 cells were treated with PDD00017273, the cells showed increased DNA damage consistent with increased γH2AX formation and reduced cell survival [25]. Inhibitor treatment induced the replication fork stalling and favored DNA repair via HR. Consequently, PDD00017273 like olaparib exhibited synthetic lethal phenotype in cells deficient in HR proteins BRCA1/2, PALB2, BARD1 etc. [96, 97]. Recently, PDD00017273 was used successfully in vitro against pancreatic ductal adenocarcinoma (PDAC) cells as a monotherapy and combination therapy [98]. However, due to poor metabolic activity, this PARG inhibitor cannot be used for cancer treatment in vivo.

We recently discovered a novel PARG inhibitor deciphering the highest potency, cell permeability and tumor cell lethality [24]. The compound known as COH34 inhibits PARG at nanomolar concentrations and induced tumor cell lethality both in vitro and in vivo. COH34 was highly potent with an IC_50_ value of 0.37 nM. COH34 mediated inhibition is highly specific to PARG and there is no-cross inhibition of other dePARylation enzymes such as TARG1 and ARH3. COH34 and PARG had a binding ratio of 1:1 wherein COH34 binds snugly into the catalytic pocket of PARG and thus competes with its normal substrate i.e. PAR. The extended PARylation in turn trapped DDR proteins (like XRCC1, APLF and CHFR) at the damaged site and thereby blocking normal DDR. COH34 exhibited synthetic lethality in cells deficient in BRCA1/BRCA1 and even those cells resistant to olaparib with its inhibitory potential exceeding olaparib. Finally, we characterized and validated the efficacy of COH34 against tumors with DDR defects in vivo. Moreover, COH34 was stable in vivo and non-toxic to mice at 20 mg/kg concentration [24]. Collectively, COH34 is a very promising lead compound for the development of dePARylation inhibitors for cancer treatment.

## Conclusion

Therapeutic targeting of PARylation and dePARylation represents an ideal target in cancer chemotherapy. Encouraged by the successful FDA approval and clinical use of olaparib, several anti-PARylation drugs are at different stages of clinical trials. Moreover, the utility of these drugs is expanding beyond breast cancer to other cancer including those in the ovary, prostate, pancreas, etc. Additional synthetic lethalities have been reported. However, the emergence of resistance against PARPi necessities the development of alternative therapeutic strategies. In this direction, a recent class of PARP1 inhibitors was developed which can degrade PARP1 (e.g. iRucaparib-AP6) with high potency and specificity [99]. However, additional studies are needed to demonstrate the therapeutic potential of these inhibitors.

DePARylation inhibitors like COH34 represent a novel class of inhibitors alternative to PARP inhibition, and may overcome chemo-resistance of PARPi. The inhibition of PARG by COH34 along with other inhibitors would be particularly effective due to the monogenic character of PARG. The possibility of resistance arising due to the redundant nature of PARP proteins could be particularly avoided with these inhibitors. Further development of dePARylation inhibitor into clinical cancer treatment may generate a huge impact on cancer patients.

## Data Availability

Not applicable.
